# Long noncoding RNA regulatory factor X3- antisense RNA 1 promotes non-small cell lung cancer via the microRNA-577/signal transducer and activator of transcription 3 axis

**DOI:** 10.1080/21655979.2022.2054910

**Published:** 2022-04-27

**Authors:** Yanjing Hu, Zhi Zhao, Gang Jin, Junhao Guo, Fangyuan Nan, Xin Hu, Yunsheng Hu, Qun Han

**Affiliations:** Department of Thoracic Surgery, The First People’s Hospital of Jiangxia District, Wuhan, Hubei, China

**Keywords:** lncRNA RFX3-AS1, non-small cell lung cancer, miR-577, signal transduction and activator of transcription 3

## Abstract

Lung cancer is the most frequent malignancy, and non-small cell lung cancer (NSCLC) is its most common pathological type. Molecular targeted therapy has been testified to be effective in intervening in the occurrence and development of malignancies. This study investigates the effect of lncRNA Regulatory Factor X3- antisense RNA 1 (RFX3-AS1) in NSCLC progression. The RFX3-AS1 profile in NSCLC tissues and cells was measured by quantitative reverse transcription PCR (qRT-PCR). The RFX3-AS1 overexpression model was constructed. The cell counting kit-8 (CCK-8) experiment and cell colony formation assay were adopted to test cell viability. The cell apoptosis was determined by flow cytometry (FCM). Cell migration and invasion were monitored by the Transwell assay, and Western blot was implemented to verify the protein profiles of signal transducer and activator of transcription 3 (STAT3), E-cadherin, Vimentin and N-cadherin. *In vivo*, we validated the impact of RFX3-AS1 overexpression on the NSCLC xenograft mouse model. The targeting relationships between RFX3-AS1 and miR-577, miR-577 and STAT3 were confirmed by the dual-luciferase reporter assay. The results manifested that overexpressing RFX3-AS1 markedly facilitated NSCLC cell proliferation, migration, invasion and epithelial-mesenchymal transition (EMT), and suppressed cell apoptosis. In contrast, miR-577, which was a downstream target of RFX3-AS1, dramatically impeded the malignant biological behaviors of NSCLC cells. STAT3 was a direct target of miR-577, and it was negatively regulated by the latter. STAT3 activation reversed miR-577-mediated anti-tumor roles. In brief, RFX3-AS1 aggravated NSCLC progression by regulating the miR-577/STAT3 axis.

## Graphical Abstract

**Figure:**
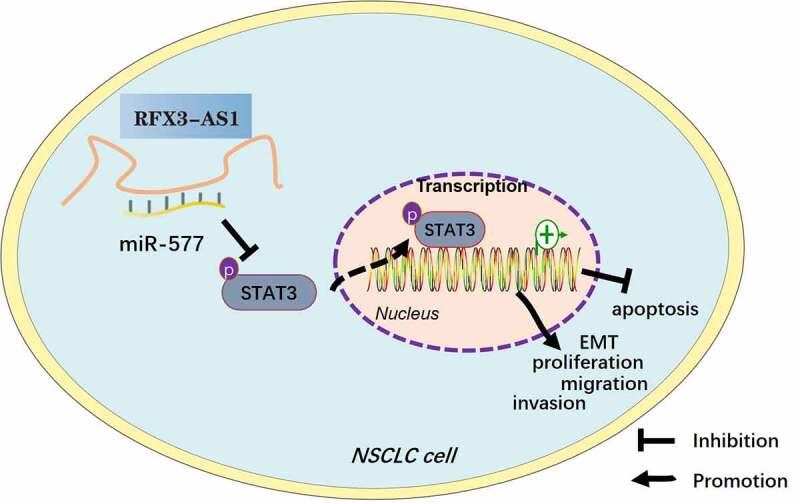

## Highlights

- lncRNA RFX3-AS1 functioned as an oncogene in NSCLC.
- miR-577 was a target of RFX3-AS1 and impeded the progression of NSCLC.
- RFX3-AS1 activated STAT3 by repressing miR-577.
- Activating STAT3 attenuated the inhibitive effect of miR-577 in NSCLC.

## 1. Introduction

Non-small cell lung cancer (NSCLC) originates from lung epithelial cells and is the most familiar pathological type of lung cancer. As the number one malignancy worldwide, NSCLC has an abysmal prognosis and poses a serious threat to people’s psychosocial lives [1,2]. For several years, NSCLC patients have been treated mainly by surgical resection, chemotherapy, and radiotherapy, but the five-year survival rate is only 19% [3]. Targeted molecular therapy, including targeting lncRNAs, is an emerging cancer therapy in NSCLC [4]. The impact of lncRNA RFX3-AS1 on the occurrence and evolution of NSCLC was studied in this paper.

Long noncoding RNAs (lncRNAs) are longer than 200 nt and do not have the protein-coding ability. However, lncRNAs mediate cell differentiation, proliferation, and cell cycle by modulating the expression of genes through diversified mechanisms. The dysregulation of lncRNA expression is the driving factor of NSCLC [5,6]. Liu et al. disclosed that PCAT1 is up-regulated in NSCLC, and PCAT1 cooperates with DKC1 to activate the VEGF/AKT/Bcl-2/caspase9 pathway, which heightens NSCLC cells’ proliferation and invasion, and dampens their apoptosis [7]. Additionally, lncRNA MCM3AP antisense RNA 1 (MCM3AP-AS1) and CBR3-AS1 are upregulated in NSCLC, and facilitate the progression of NSCLC by activating different pathways [8,9]. lncRNA RFX3-AS1 is an anti-coding strand for the RFX-coding gene located on chromosome 9p24.2. Scholars have discovered that RFX3-AS1 is highly expressed in breast cancer cells, and overexpressing RFX3-AS1 hampers cell apoptosis and enhances cell viability [10]. Nonetheless, the function of RFX3-AS1 has been rarely studied in NSCLC.

MicroRNAs (miRNAs) are noncoding RNAs of about 20–25 nucleotides in length, and their dysregulation is strongly linked with tumor growth, differentiation, and metastasis. Also, miRNAs contribute to pathological processes such as apoptosis, inflammation, and oxidative stress [11]. Some scholars revealed that miR-101-3p modulates the sensitivity of NSCLC cells to cisplatin by regulating ATG4D-mediated cell autophagy [12]. Besides, studies by Gong et al. displayed that Fentanyl chokes the proliferation and invasion of NSCLC cells by up-regulating miR-331-3p and hampering HDAC5 [13]. miR-577 has two sides in tumors. On one hand, overexpressing miR-577 notably represses the malignant biological behaviors of pancreatic cancer cells [14]. On the other hand, miR-577 is up-regulated in gastric cancer, and it targets serum deprivation protein response to facilitate the TGF-β signal transduction, thereby inducing gastric cancer metastasis and chemoresistance [15]. In this paper, the effects and specific mechanisms of miR-577 in NSCLC were studied in detail.

Signal transduction and activator of transcription 3 (STAT3) is a core member of the STAT family, which is considered as a key regulator of cancer and involved in tumor invasion, metastasis, angiogenesis, and immune escape [16]. STAT3 is strongly linked with the development of NSCLC. For example, up-regulating miR-196b-5p activates the STAT3 signaling and heightens NSCLC cell growth by dampening the FAS expression [17]. As another example, Zoledronic weakens epithelial-mesenchymal transition (EMT) and restores the chemosensitivity of NSCLC to gefitinib by inactivating the IL-6/JAK/STAT3 pathway [18]. Additionally, Zheng et al. testified that the use of the STAT3 inhibitor W2014-S suppresses NSCLC cell proliferation, migration and invasion and strengthens chemoresistance to gefitinib [19]. These findings hint that inhibiting STAT3 activation helps to treat NSCLC.

In a nutshell, a novel lncRNA, RFX3-AS1, has been confirmed to be up-regulated and to boost adverse biological behaviors in breast cancer. Nevertheless, its function in NSCLC remains unknown Meanwhile, miR-577 has been reported to have both pro- and anti-carcinogenic effects and STAT3 expedites the progression of NSCLC. Fortunately, our experimental results manifested that RFX3-AS1 was up-regulated in NSCLC tissues and cells, with a targeting relationship being established between it and miR-577. Hence, we hypothesized that RFX3-AS1 functions as an oncogene in NSCLC and facilitates NSCLC progression by modulating the miR-577/STAT3 axis. In conclusion, this study may offer theoretical reference and academic support for NSCLC treatment.

## 2 Materials and methods

### 2.1 Clinical samples

In the current research, the clinical samples were collected from 32 NSCLC patients who underwent surgical resection in Wuhan Jiangxia first people’s Hospital from May 2015 to November 2015, and their tumor tissues and paracancerous normal tissues (more than 5 cm away from the lesion) were collected. All tissues were examined pathologically and confirmed by the head of the Department of Pathology of Wuhan Jiangxia first people’s Hospital. The tissues were snap-frozen and stored in liquid nitrogen at −196°C. Prior to the surgery, none of the above patients had undergone adjuvant treatment such as radiotherapy, and all of them had signed informed consent forms. This experiment was granted by the Ethics Committee of Wuhan Jiangxia first people’s Hospital. The clinical information of the 32 NSCLC patients is displayed in Table 1.

**Table 1.** The association between clinical parameters and RFX3-AS1 level

Variable	RFX3-AS1 Expression		P-value
	Low (n = 11)	High (n = 21)	
Age ≤60 > 60	5 6	7 14	0.501
Gender Male Female	3 8	12 9	0.108
Tumor size ≤3 cm > 3 cm	7 4	8 13	0.169
TNM I–II III–IV	8 3	7 14	0.034 *
Lymph node metastasis Positive Negative	2 9	13 8	0.019 *

* P < 0.05 signified the statical significance.

### 2.2 Cell culture

Human normal lung epithelial cell lines HSC-2 and NSCLC cell lines (HCC15, PC-9, Y-803, and A549) were ordered from the American Type Culture Collection (ATCC, Rockville, MD, USA). The above cells (1 × 10^5^ cells/mL) were inoculated in the RPMI-1640 medium comprising 10% inactivated newborn fetal bovine serum (FBS, Hyclone, Logan, UT, USA) and cultured at 37°C with 5% CO_2_ [20].

### 2.3 Cell transfection

PC-9 and A549 cell lines were inoculated in 12-well plates at the adjusted concentration of 5 × 10^5^ cells/well and cultured with antibody-free RPMI-1640 containing 10% fetal bovine serum (FBS). RFX3-AS1 overexpression plasmids and corresponding negative controls were transfected into cells using the FuGene®HD Transfection Reagent (Roche, Shanghai, China) following manufacturer’s protocol. miR-577 mimics and miR-577 negative control (miR-NC) were transfected into the cells using the same method as above. In addition, the cells were treated with the STAT3 agonist colivelin (Cat.No. sc-361,153, Santa Cruz Biotechnology, California, USA) as required. After incubation for 48 hours, the cells were harvested and the expression of RNA or protein was determined by quantitative reverse transcription PCR (qRT-PCR) and Western blot (WB).

### 2.4 Quantitative reverse transcription PCR (qRT-PCR)

PC-9 and A549 cell lines were taken, and total cellular RNA was isolated with the TRIzol reagent (Invitrogen, Carlsbad, CA, USA). Total RNA purity was tested. The RevertAid First Strand cDNA Synthesis Kit (Thermo Fisher Scientific, Waltham, MA, USA) was employed to reversely transcribe the total RNA into cDNA. Then, TOYOBO SYBR Green Realtime PCR Master Mix (TOYOBO, Osaka, Japan) was applied for qRT-PCR. Glyceraldehyde-3-phosphate dehydrogenase (GAPDH) was used as the endogenous control of RFX3-AS1, and U6 was that of miR-577. The primer sequence of each molecule in the study is exhibited in Table 2.

**Table 2.** The primer sequence in PCR

Gene	Sequences	
RFX3-AS1	Forward	5’-TCCTGGGTCCCTAAGTCGTA-3’
	Reverse	5’-AGAAGGCTTTGCTCTGTTGC-3’
miR-577 STAT3	Forward	5’- GGACUUUCUUCAUUCACACCG −3’
	Reverse Forward Reverse	5’- GACCACUGAGGUUAGAGCCA −3’ 5’- AGAAGGAGGCGTCACTTTCA-3’ 5’- TTTCCGAATGCCTCCTCCTT-3’
GAPDH	Forward	5’- TGGTTGAGCACAGGGTACTT −3’
	Reverse	5’- CCAAGGAGTAAGACCCCTGG −3’
U6	Forward	5’- CGCTTCGGCAGCACATAT −3’
	Reverse	5’- AAATATGGAACGCTTCACGA −3’

### 2.5 Cell counting kit (CCK-8) assay

CCK-8 assay was utilized to test the viability of PC-9 and A549 cell lines. The stably transfected cells were seeded into 96-well plates (1 × 10^3^ cells/well) and incubated for 24 hours. 10 μL of CCK-8 reagent (Dojindo Molecular Technologies, Kumamoto, Japan) was added to each well as per the manufacturer’s instructions and incubated for 0, 24, 48, 96 hours as required for the experiments. The optical density at 450 nm was gauged with a microplate reader (Bio-Tek Instruments, Winooski, VT, USA) [21].

### 2.6 Cell colony formation experiment

After stable cell transfection, the cells were subjected to concentration adjustment and seeded at 200 cells per well in 6-well plates (2 mL culture medium per well). The cells were cultured at 37°C for 10 ~ 14 days, and the culture was terminated when colonies were visible to the naked eye. Subsequently, the cells were immobilized with 4% paraformaldehyde for 15 minutes at room temperature (RT) and dyed with 0.2% crystal violet for 5 minutes. A colony was counted if it contained more than 50 cells. Colony formation rate = the colony formation number/the inoculated cell number×100% The experiment was implemented three repeated times.

### 2.7 Flow cytometry (FCM)

PC-9 and A549 cells were transfected for 24 hours, trypsinized, and then collected. The cell density was adjusted to 2 × 10^5^ cells/well, and the cells were inoculated in 6-well plates. After 24 hours of culture, the cells were cleaned twice with PBS and suspended in the annexin-binding buffer. Next, 5 μL AnnexinV-FITC and 5 μL PI were added to the cell suspension, mixed well and maintained at RT for 15 minutes. Stained cells were observed by FACS Calibur Flow Cytometer (BD Biosciences, SanJose, CA, USA) as well as the CellQuest software (BD Biosciences) and analyzed for apoptosis [22].

### 2.8 Transwell assay

Transwell assay was conducted to test cell migration and invasion. After transfection for 48 hours, PC-9 and A549 cell lines were dispersed with 0.25% trypsin, centrifuged, resuspended, and cultured in 24-well plates. Matrigel pre-coated chambers (8 µM poresize, Corning, Beijing, China) were utilized in the invasion experiment. 5 × 10^4^ cells were put in the upper chamber, and the medium supplemented with 400 μL RPMI-1640 medium (containing 20% FBS) was placed in the lower chamber. After incubation at RT for 24 hours, the cells in the upper chamber were removed with a cotton swab. The transwell membranes were secured with 4% paraformaldehyde for 10 minutes and dyed with 0.5% crystal violet. After the membranes were flushed with tap water, cell numbers were calculated with an inverted microscope (magnification: ×200; Olympus Corporation). All other steps of the migration experiment were the same as the invasion experiment except that Matrigel was not used [23].

### 2.9 Western blot (WB)

Total cellular proteins of PC-9 and A549 cells were isolated using the protein lysate (Roche). The BCA Protein Assay Kit (Beyotime Institute of Biotechnology) was employed to verify the protein content. 50 μg total protein was subjected to 12% polyacrylamide gel and went through 2 hours of electrophoresis at 100 V. Then, the protein was electrically transferred to polyvinylidene difluoride (PVDF) membranes, which were blocked with 5% skimmed milk at RT for 1 hour and incubated overnight at 4°C with the following primary antibodies (1:1000, Abcam, USA): Anti-E-cadherin antibody (ab231303), Anti-Vimentin antibody (ab20346), Anti-N-Cadherin antibody (ab76011), Anti-STAT3 (phospho S727) antibody (ab32143), Anti-STAT3 antibody (ab68153), Anti-Bcl2 antibody (ab32124), Anti-Bax antibody (ab32503), Anti-Caspase3 antibody (ab32351), and Anti-GAPDH antibody (ab245356). After the membranes were rinsed with TBST, they were incubated with horseradish peroxidase (HRP)-tagged anti-rabbit secondary antibody (concentration: 1:300) for 1 hour. Finally, the bands were visualized with the electrochemiluminescence automatic chemiluminescence imaging analysis system (Tanon, Shanghai, China) [24].

### 2.10 Tumor formation in nude mice

BALB/c nude mice (4–6 weeks old) were acquired from the Animal Experimental Center of Huazhong University of Science and Technology (Wuhan, China). They were kept in a specific pathogen-free environment for one week, with 12 hours of light/dark cycle and sufficient food and water. A549 cells transfected with RFX3-AS1 overexpression plasmids were selected, and the cell concentration was adjusted to 1 × 10^7^ mL^−1^. Then, 0.1 mL of cell suspension was injected into the left axilla of each nude mouse subcutaneously. Within four weeks after the injection, the vitality, body weight and survival status of the mice were monitored. Subcutaneous tumor volume (V) was determined and recorded with calipers every four days from the twelfth day. V = 0.5× long diameter×short diameter^2^. The mice were euthanized on the 28^th^ day, and the xenograft tumors were dissected and weighed. Immunohistochemistry was conducted for gauging KI67, E-cadherin, Vimentin, and STAT3 (phospho S727) in the tumor tissues. The primary antibodies include KI67 (ab15580), E-cadherin (ab1416), Vimentin (ab8069), and p-STAT3 (ab32143) all of which were purchased from Abcam (USA). All animal procedures were endorsed by the Medical Ethics Committee of Wuhan Jiangxia first people’s Hospital [25].

### 2.11 Dual-luciferase reporter assay

RFX3-AS1-WT, RFX3-AS1-MT, STAT3-WT and STAT3-MT were obtained from Promega Corporation (Madison, WI, USA). The above luciferase reporter gene plasmids were individually transfected into the 293 T cells. Meanwhile, miR-577 mimics or miR-NC were transfected into 293 T cells, respectively. The cells were incubated for 48 hours and then rinsed with PBS, and the luciferase activity was tested by the Dual-luciferase Reporter Assay kit (Promega Corporation) [26].

### 2.12 Statistical analysis

Statistical Software GraphPad Prism 8 (GraphPad Software, Inc., City, State) was employed to analyze the data, and the results were presented as mean ± SD (x ± s). Differences between multiple groups of data were analyzed by one-way ANOVA, and differences between two groups of data were compared by Tukey post hoc test analysis. *P* < 0.05 represented statistical significance.

## 3 Results

We tested RFX3-AS1 level in NSCLC tissues and cells for verifying the characteristic expression in NSCLC. Next, gain-of functional assays of RFX3-AS1 were performed for confirming its role in mediating the proliferation, migration, invasion and apoptosis of NSCLC cells. The downstream targets of RFX3-AS1 were then predicted and confirmed.

### 3.1 RFX3-AS1 was highly expressed in NSCLC

**Figure 1.**
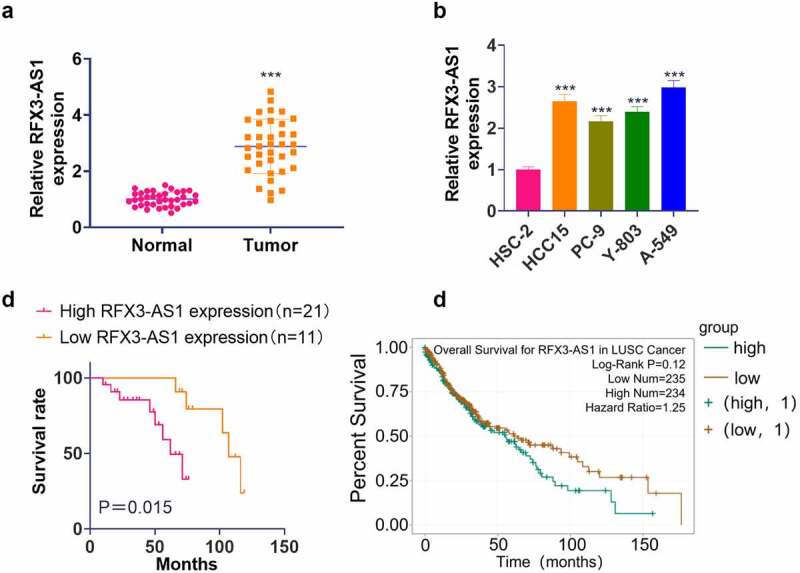
RFX3-AS1 was highly expressed in NSCLC. NSCLC tissues and paired adjacent normal tissues were harvested. Human normal lung epithelial cell line HSC-2 and NSCLC cell lines (HCC15, PC-9, Y-803, and A549) were routinely cultured, and sample patients were closely followed up.A: The RFX3-AS1 profile in NSCLC patients was examined by qRT-PCR. ***P < 0.001 (vs. Normal group); B: The profiles of RFX3-AS1 in HSC-2, HCC15, PC-9, Y-803 and A549 cell lines were compared by qRT-PCR. ***P < 0.001 (vs. HSC-2 group) N = 3. C: The overall survival of NSCLC patients with a high or low level of RFX3-AS1 was analyzed using a K-M plotter assay. D: Starbase database was applied to query the association between the RFX3-AS1 expression and the survival prognosis of NSCLC patients.

qRT-PCR outcomes illustrated that the RFX3-AS1 profile in NSCLC tissues and cells was distinctly higher than that in adjacent normal lung tissues and lung epithelial cell line HSC-2 (*P* < 0.05, Figure 1a-b). Besides, our K-M plotter analysis exhibited that patients with lower RFX3-AS1 expression had better overall survival than those with higher RFX3-AS1 expression (*P* = 0.015, Figure 1c). Additionally, the Starbase database (http://starbase.sysu.edu.cn/) revealed that the overall survival of patients with high RFX3-AS1 expression was much lower than those with low RFX3-AS1 expression (*P* = 0.12, Figure 1d). These findings manifested that RFX3-AS1 might act as an oncogene in NSCLC.

### 3.2 Overexpressing RFX3-AS1 expedited NSCLC progression

**Figure 2.**
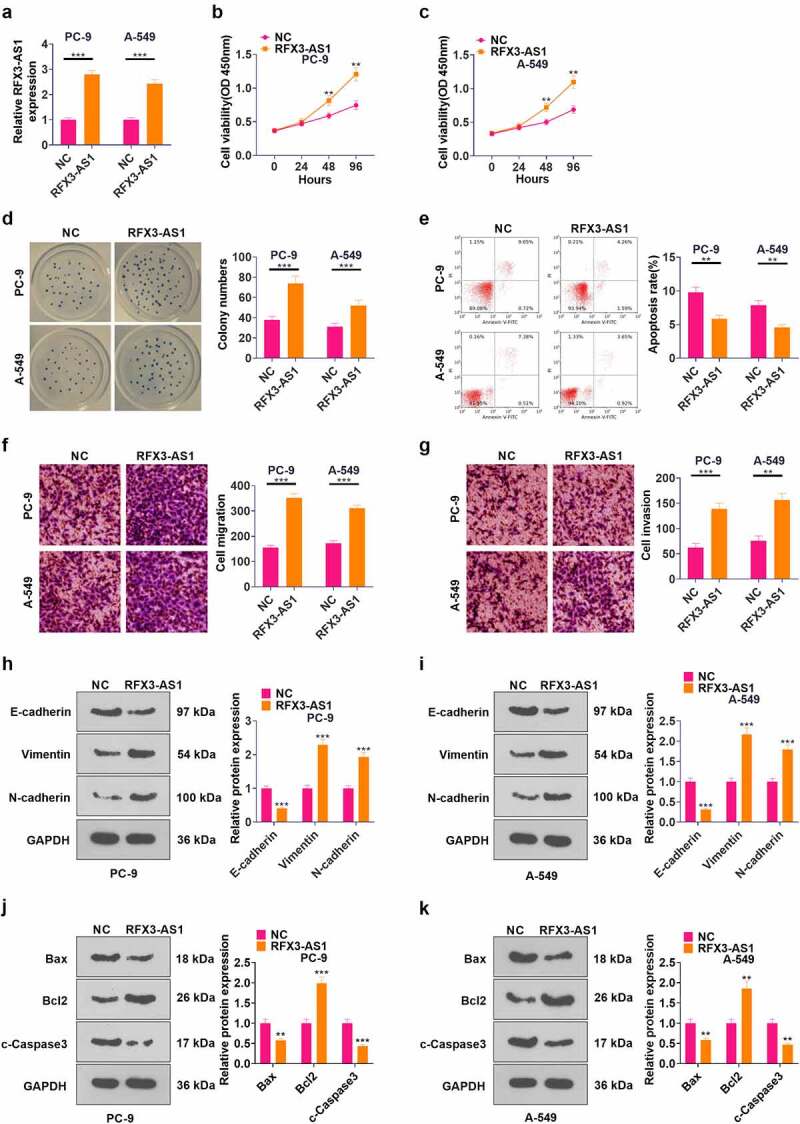
Overexpressing RFX3-AS1 promoted the progression of NSCLC. The RFX3-AS1 overexpression model was constructed in PC-9 and A549 cell lines.A: The RFX3-AS1 expression was assessed by qRT-PCR. B-C: Cell proliferation was monitored by CCK-8 after overexpressing RFX3-AS1. D: Cell colony formation ability was analyzed by the cell colony formation assay. E: Cell apoptosis was assayed by FCM. F-G: Transwell assay was conducted to evaluate cell migration and invasion. H-I: The protein profiles of E-cadherin, Vimentin and N-cadherin were compared by WB. J-K: The protein profiles of Bcl2, Bax and Caspase3 were compared by WB. *P < 0.5, **P < 0.01, ***P < 0.001 (vs.NC group) N = 3.

The RFX3-AS1 overexpression model was constructed in PC-9 and A549 cell lines (*P* < 0.05, Figure 2a). CCK-8 assay was employed to test cell proliferation, which revealed that overexpressing RFX3-AS1 markedly heightened NSCLC cell proliferation (*P* < 0.05, Figure 2b-c). Cell colony formation experiment results uncovered that RFX3-AS1 overexpression enhanced cell viability (*P* < 0.05, Figure 2d). FCM demonstrated that up-regulation of RFX3-AS1 repressed cell apoptosis (*P* < 0.05, Figure 2e). The data from Transwell assay testified that overexpressing RFX3-AS1 boosted tumor cell migration and invasion (*P* < 0.05, figure 2f-g). Furthermore, the expression of EMT markers (including E-cadherin, Vimentin, and N-cadherin) was compared by WB. As a result, overexpressing RFX3-AS1 down-regulated E-cadherin, and up-regulated Vimentin and N-cadherin (*P* < 0.05, Figure 2h-i). WB results testified that compared with the NC group, the RFX3-AX1 group has enhanced Bcl2 and reduced Bax and c-Caspse3 levels (Figure 2j-k). Hence, overexpression of RFX3-AS1 aggravated malignant biological behaviors of NSCLC cells.

### 3.3 Overexpressing RFX3-AS1 heightened tumor growth in nude mice

**Figure 3.**
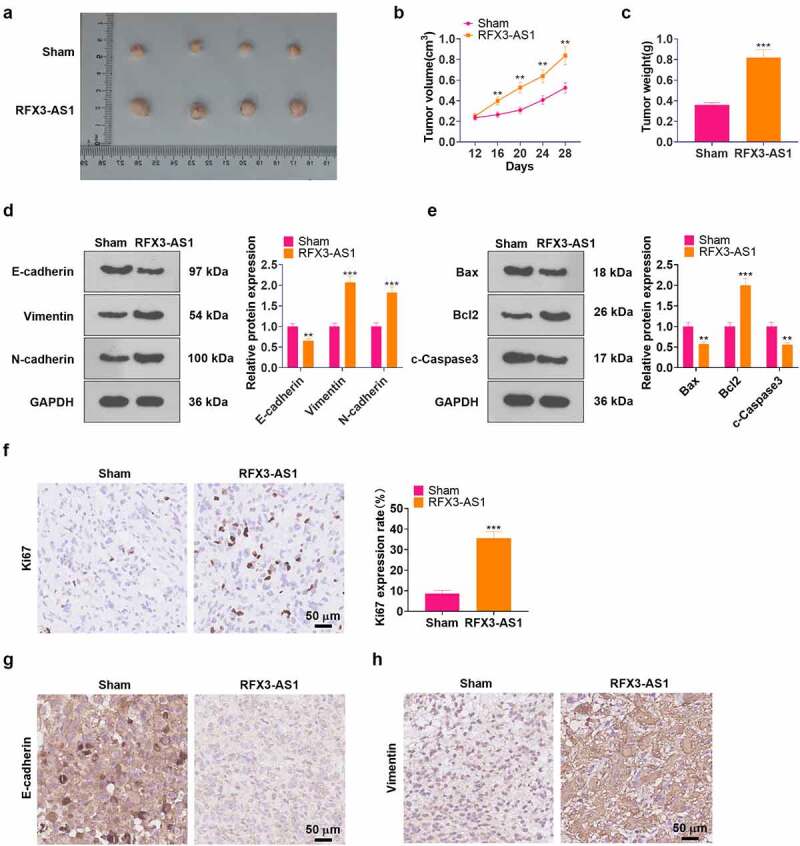
Overexpressing RFX3-AS1 facilitated tumor growth in nude mice. A549 cells were transfected with RFX3-AS1 overexpression plasmids, and 0.1 mL cell suspension (1 × 10^7^ cells ml^−1^) was injected into the mice’s left axilla subcutaneously, C: A tumor xenograft model was conducted to observe the influence of overexpressing RFX3-AS1 on tumors *in vivo*, and tumor volume and data were recorded. D-E: The profiles of E-cadherin, Vimentin, N-cadherin, Bcl2, Bax and Caspase3 in the tumor tissues were examined by WB. F-H. IHC was performed for detecting KI67 (F), E-cadherin (G), and Vimentin (H) in the tumor tissues. Scale bar = 50 μm. **P < 0.01, ***P < 0.001(vs.Sham group) N = 5.

*In-vivo* experiments of RFX3-AS1 were performed for evaluating the role of RFX3-AS1 in NSCLC cell growth. The data displayed that overexpressing RFX3-AS1 evidently accelerated tumor growth in nude mice (*P* < 0.05, Figure 3a-c). As testified by WB results, overexpressing RFX3-AS1 up-regulated Vimentin, N-cadherin, and Bcl2 while down-regulated E-cadherin, Bax, and c-Caspase3 in the formed tumor tissues (*P* < 0.05, Figure 3d-e). The proliferation marker, KI67, as well as EMT markers, including E-cadherin and Vimentin, were gauged by IHC. It was found that RFX3-AS1 overexpression elevated the KI67-positive cell rate and strengthened Vimentin expression, while it choked the E-cadherin profile in the RFX3-AS1 group (figure 3f-h). These findings hinted that overexpressing RFX3-AS1 augmented the growth of NSCLC cells *in vivo*.

### 3.4 RFX3-AS1 targeted miR-577

**Figure 4.**
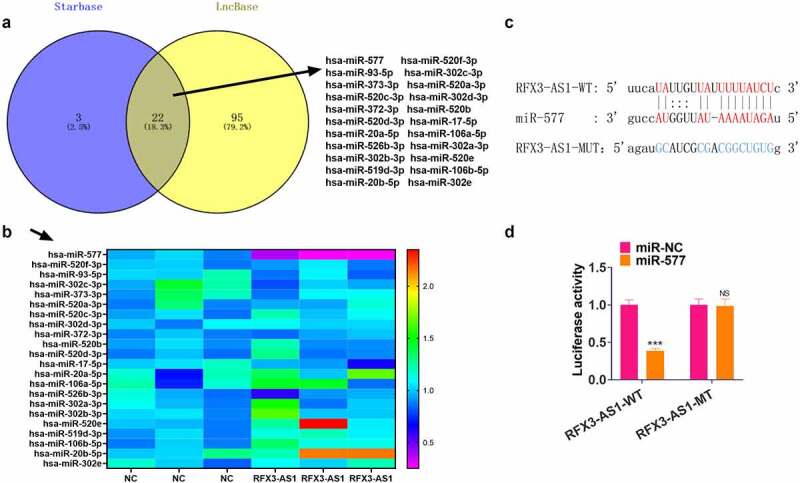
RFX3-AS1 targeted miR-577. A. The miRNA targets of RFX3-AS1 were searched through two online databases, including Starbase and LncBase v.2. The Venny’s diagram was utilized for analyzing the common miRNA targets of the two databases. The results exhibited that 22 miRNAs were potential targets of RFX3-AS1. B. qRT-PCR was performed for evaluating the 22 miRNAs in A549 cells transfected with RFX3-AS1 overexpression plasmids. C. The binding sites between RFX3-AS1 and miR-577 were shown. D. The dual-luciferase reporter assay was performed in 293 T cells to verify the binding relationship between miR-577 and RFX3-AS1. NS *P* > 0.05, ***P < 0.001(vs.miR-NC group) N = 3.

For investigating the downstream mechanism of RFX3-AS1, we searched the miRNA targets of RFX3-AS1 through two online databases, including Starbase and LncBase v.2. The Venny’s diagram was adopted for analyzing the common miRNA targets of the two databases. Our discovery was that 22 miRNAs were potential targets of RFX3-AS1 (Figure 4a). We performed qRT-PCR for evaluating the 22 miRNAs in A549 cells transfected with RFX3-AS1 overexpression plasmids. The data disclosed that miR-577 exhibited the most pronounced down-regulation (Figure 4b). For confirming the binding association between RFX3-AS1 and miR-577, we implemented a dual-luciferase reporter assay in 293 T cells (Figure 4c). As a result, miR-577 mimics markedly hindered the luciferase activities of 293 T cells transfected with RFX3-AS1-WT (compared with the miR-NC group), while they had no significant impact on the luciferase activities of 293 T cells transfected with RFX3-AS1-MUT (Figure 4d). Hence, we concluded that RF3X-AS1 binds with and downregulates miR-577.

### 3.5 Transfection of miR-577 mimics hampered NSCLC evolvement

**Figure 5.**
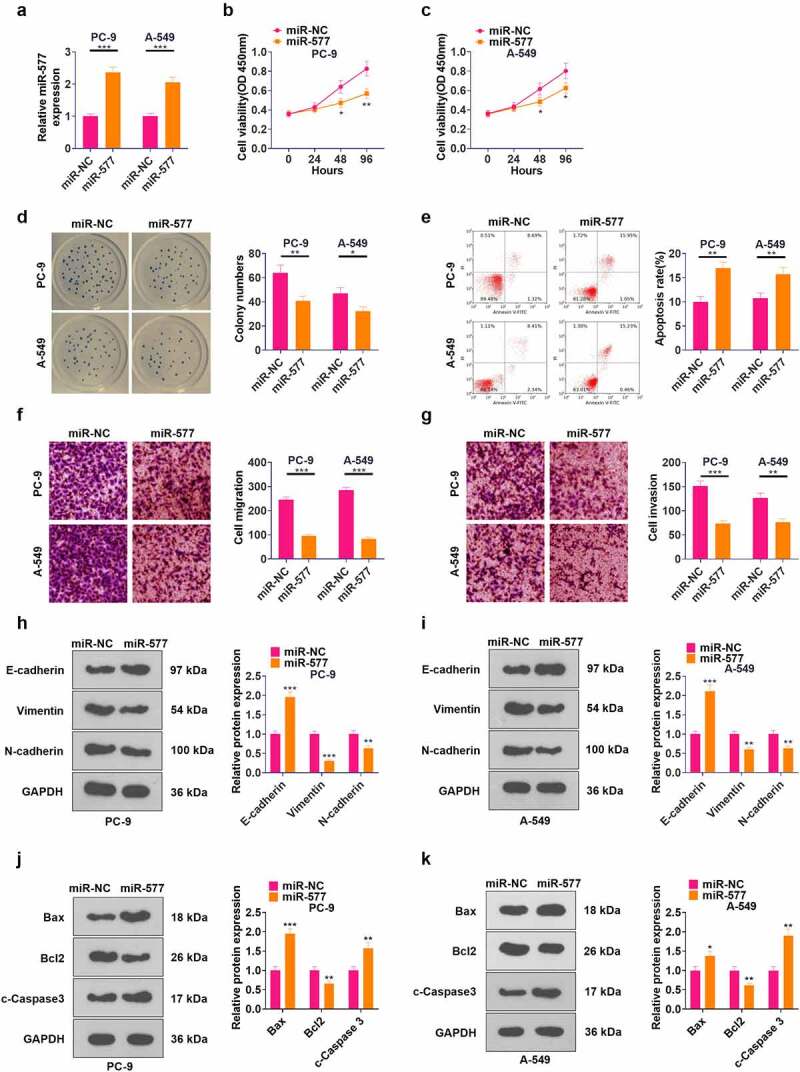
Transfection of miR-577 mimics significantly suppressed the progression of NSCLC. miR-577 mimics were transfected in PC-9 and A549 cell lines. A: The miR-577 profile was tested by qRT-PCR. B-C: The CCK-8 assay was implemented to examine cell proliferation after transfection with miR-577. D: Cell viability was measured by the cell colony formation assay. E: The apoptotic rate was analyzed by FCM. F-G: Transwell assay was applied to determine cell proliferation and invasion. H-I: The profiles of E-cadherin, Vimentin and N-cadherin were examined by WB. J-K: The protein profiles of Bcl2, Bax and Caspase3 were compared by WB. *P < 0.5, **P < 0.01, ***P < 0.001(vs.miR-NC group) N = 3

To probe the function of miR-577 in NSCLC, we transfected miR-577 mimics in PC-9 and A549 cell lines (*P* < 0.05, Figure 5a). CCK-8 and cell colony formation assay results testified that the cell viability was abated following transfection of miR-577 mimics (*P* < 0.05, Figure 5b-d). FCM results illustrated that the miR-577 mimic transfection notably elevated the apoptotic rate of cells (*P* < 0.05, Figure 5e). As revealed by Transwell assay results, transfection of miR-577 mimics hampered cell migration and invasion (*P* < 0.05, figure 5f-g). The profiles of EMT markers and apoptosis-related proteins were monitored by WB, which uncovered that the miR-577 mimic transfection up-regulated E-cadherin, Bax, and c-Caspase3, and down-regulated Vimentin, N-cadherin, and Bcl2 (*P* < 0.05, Figure 5h-k). These findings manifested that miR-577 restrained the development of NSCLC *in vitro*.

### 3.6 RFX3-AS1 targeted miR-577 to up-regulate STAT3

**Figure 6.**
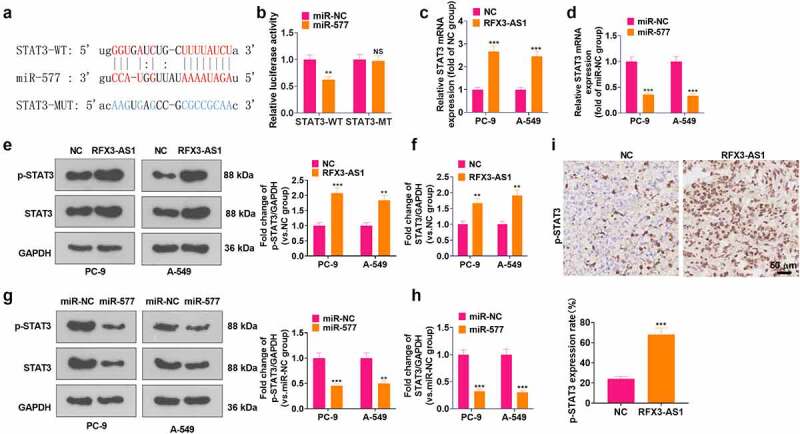
RFX3-AS1 targeted miR-577 to regulate STAT3. A: Starbase database was adopted for analysis of base pairing between miR-577 and STAT3. B: Dual-luciferase reporter assay was performed in 293 T cells transfected with STAT3-WT or STAT3-MT, with miR-NC or miR-577 mimics. C-D: The p-STAT3/STAT3 profile after overexpression of RFX3-AS1 or transfection of miR-577 mimics was monitored by WB. E. IHC was conducted for detecting p-STAT3 in the tumor tissues shown in Figure 3. NS*P* > 0.05, **P < 0.01, ***P < 0.001(vs.miR-NC or NC group). N = 3.

A query of the online website Starbase displayed that there are base binding complementation sites between miR-577 and STAT3 (Figure 6a). Then, a dual-luciferase reporter assay was conducted for verification. It turned out that the transfection of miR-577 mimics impeded the luciferase activity of STAT3-WT-transfected cells, but it had no evident inhibitory effects on STAT3-MT (*P* > 0.05, Figure 6b). RT-PCR data supported that RFX3-AS1 upregulation enhanced STAT3 mRNA expression, whereas miR-577 mimics reduced STAT3 mRNA level (*P* < 0.05, Figure 6c,d). Additionally, WB outcomes testified that overexpressing RFX3-AS1 fostered the phosphorylation and total protein of STAT3, while transfection of miR-577 mimics inactivated STAT3 pathway (*P* < 0.05, Figure 6e-h). In addition, STAT3 phosphorylation in the tumor tissues was heightened by RFX3-AS1 overexpression (Figure 6i). These findings manifested that RFX3-AS1 targeted miR-577 and thus activated STAT3.

### 3.7 Activation of STAT3 curbed the tumor-suppressive effect of miR-577 in NSCLC

**Figure 7.**
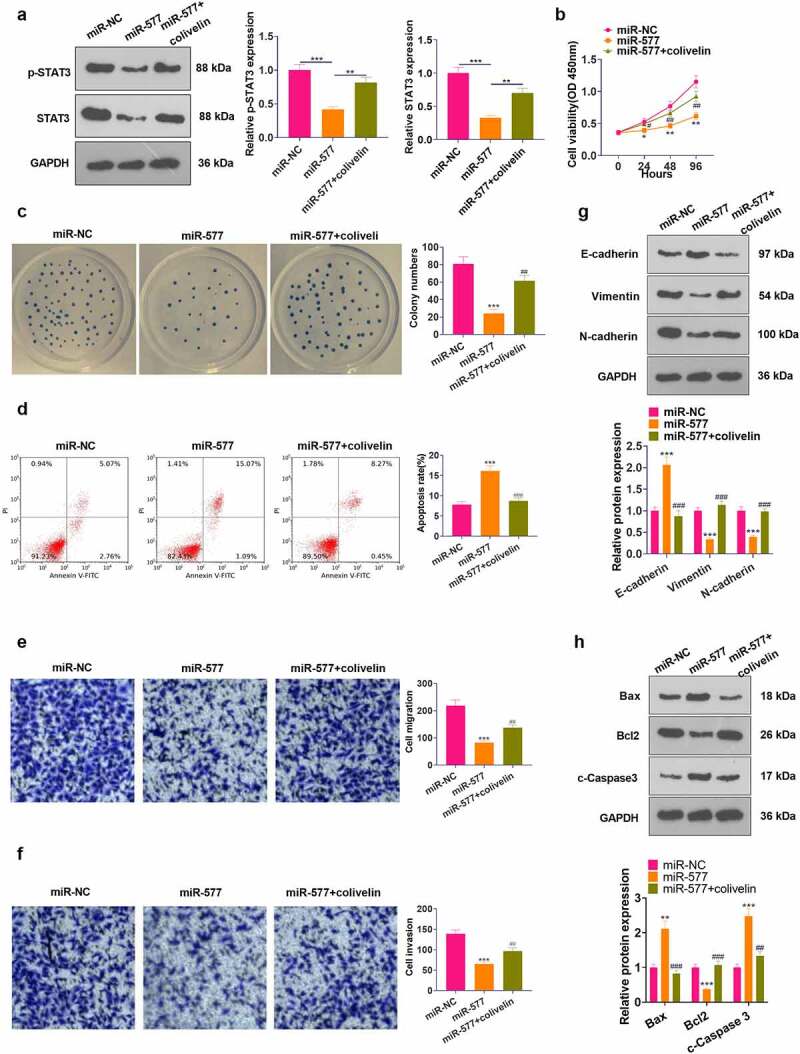
Activating STAT3 weakened the tumor-suppressive effect of miR-577 in NSCLC. PC-9 cells were transfected with miR-577 mimics or interfered with colivelin (50 µg/mL) for 12 hours. A: WB was performed to verify the STAT3 expression after each factor treatment. B: CCK-8 was utilized to testify cell proliferation. C: Cell viability was analyzed by the cell colony formation assay. D. Cell apoptosis was analyzed by FCM. E-F: Transwell assay was implemented to monitor cell migration and invasion. G: Protein expression of E-cadherin, Vimentin and N-cadherin was examined by WB. The protein profiles of Bcl2, Bax and Caspase3 were compared by WB. *P < 0.5, **P < 0.01, ***P < 0.001 (vs.miR-NC group); #*P* < 0.5, ##*P* < 0.01, ###*P* < 0.001(vs.miR-577 group) N = 3.

To confirm that RFX3-AS1 regulated the miR-577/STAT3 axis to mediate NSCLC development, we examined the influence of STAT3 on NSCLC cells. First, we transfected miR-577 mimics into PC-9 cells. Then, we applied the STAT3 agonist colivelin in the cells. The expression of STAT3 was verified by WB, which revealed that colivelin treatment signally abated STAT3 inactivation induced by miR-577 mimics (*P* < 0.05, Figure 7a). CCK-8, cell colony formation assay, FCM, Transwell assay and WB were applied to determine cell viability, apoptosis, migration, invasion and EMT, respectively. As a result, the transfection of miR-577 mimics impeded the viability, migration, invasion and EMT of tumor cells and heightened apoptosis. Nevertheless, the use of colivelin partially eliminated the tumor-suppressive effect of miR-577 (*P* < 0.05, Figure 7b-h). Thus, the activation of STAT3 reversed miR-577-mediated anti-tumor effects in NSCLC.

## 4 Discussion

**Figure 8.**
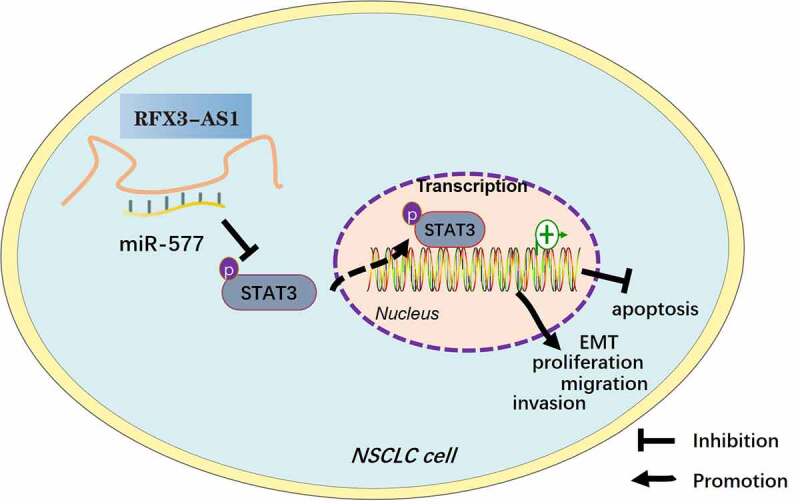
Graphical abstract. Overexpression of RFX3-AS1 targets and inhibits miR-577 expression, thereby activating STAT3, which promotes NSCLC cell proliferation, viability, migration, invasion, and EMT, and dampens apoptosis.

Though the incidence of lung cancer has declined in recent years, lung cancer remains the leading contributor to cancer-related deaths worldwide [27]. In addition, the overall survival of NSCLC remains poor due to low early diagnosis rate, metastasis, and recurrence [28]. lncRNAs are considered potential targets for cancer and can be involved in the development of NSCLC as oncogenes or tumor suppressor genes [29]. We experimentally studied RFX3-AS1 and observed that it was highly expressed in NSCLC and was closely related to the poor prognosis of NSCLC (Figure 8).

lncRNAs interact with DNA or proteins and thus participate in various pathophysiological processes. Multiple altered lncRNAs have been found to be potential prognostic markers for NSCLC [30,31]. Targeting lncRNAs suppresses the progression of NSCLC. Thus, lncRNAs function as potential therapeutic targets for NSCLC [32–38]. RFX3-AS1 is a novel oncogene that is still under-researched. Hou et al. exhibited that overexpression of RFX3-AS1 elevates the proliferation and impedes apoptosis of breast cancer cells [9], indicating that RFX3-AS1 is a potential oncogene in tumors. Here, we confirmed that enhanced RFX3-AS1 level predicts poorer survival of NSCLC patients. Overexpressing RFX3-AS1 enhanced NSCLC cells’ proliferation, migration, invasion and EMT and attenuated cell apoptosis *in vitro. In vivo*, RFX3-AS1 accelerated tumor growth. Besides, patients with high RFX3-AS1 expression had a worse survival status than those with low RFX3-AS1 profiles. These results imply that RFX3-AS1 acts as an oncogene in NSCLC.

Metastasis is a major challenge in treating NSCLC. Emerging studies have supported that miRNAs have potent effects in mediating NSCLC metastasis [39]. miRNAs have been found to mediate NSCLC growth, angiogenesis, EMT, and immune escape [40–43]. Moreover, numerous studies have suggested that miRNAs have great potential in the diagnosis, prognosis and treatment of NSCLC [44,45]. miR-577 is an emerging noncoding RNA whose oncogenic effects have been corroborated in many studies. Yin et al. stated that miR-577 targets and curbs Rab25 expression, thereby suppressing metastasis and EMT in breast cancer cells [46]. Xue et al. revealed that miR-577 targets SphK2 to abate the proliferation, migration and invasion of pancreatic cancer cells [47]. Also, SNHG3 targets miR-577 to up-regulate SMURF1 and heighten the development of prostate cancer [48]. Fortunately, we confirmed through experiments that miR-577 is an important downstream target of RFX3-AS1, and the transfection of miR-577 mimics significantly mitigates the malignant biological behaviors of NSCLC cells. These findings indicate that miR-577 is a promising curative target to suppress the progression of NSCLC.

lncRNAs can affect the translation of mRNAs by interacting with miRNAs. Our results also confirm that RFX3-AS1 activates STAT3 by targeting miR-577. STAT3 interacts with the SH2 domain to form a homodimer or heterodimer, which is heterotopic to the nucleus to regulate gene transcription [49]. STAT3 is activated in multiple malignancies. Lin et al. confirmed that IL-6 is overexpressed in colorectal cancer and activates STAT3, thereby facilitating the proliferation, cell cycle and angiogenesis of tumor cells [50]. Wang et al. demonstrated that Amygdalin reduces the phosphorylation of JAK2 and STAT3 and the levels of IFN-γ and TNF-α in HBV-associated liver cancer, thereby weakening the viability, invasion and migration of tumor cells [51]. Other reports have illustrated that RHPN2 activates the STAT3 pathway and expedites the progression of ovarian cancer, and the use of the STAT3 inhibitors signally eases the malignant cell behaviors induced by RHPN2 [52]. More importantly, STAT3 activation induces a poor prognosis for NSCLC, and inhibition of STAT3 has great potential for treating NSCLC [53,54]. Additionally, AK027294 enhances the growth of NSCLC by up-regulating STAT3 [55]. Peng et al. disclosed that overexpressing LINC81507 competitively dampens miR-199b-5p, induces the up-regulation of CAV1 and inactivation of STAT3, and restrains the malignant cell behaviors in NSCLC [56]. Our findings implied that miR-577 targeted and inactivated STAT3, and the use of the STAT3 agonist colivelin restrained the inhibition of miR-577 on NSCLC cells, including partially recovering tumor cell proliferation, migration, invasion and EMT. Thus, activating STAT3 boosts the development of NSCLC.

## Conclusion

In conclusion, RFX3-AS1 was up-regulated in NSCLC, and RFX3-AS1 targeted miR-577 to activate STAT3, thereby aggravating NSCLC. This study revealed a novel regulatory axis, namely the RFX3-AS1-miR-577-STAT3 axis, in NSCLC. Nonetheless, diversified clinical samples should be further added to increase supportive evidence for the possibility of RFX3-AS1 in the diagnosis of NSCLC, and the regulatory effect of the RFX3-AS1-miR-577-STAT3 axis needs to be confirmed in more animal experiments.

## Data Availability

The data sets used and analyzed during the current study are available from the corresponding author on reasonable request.

## Abbreviations

CCK-8: cell counting kit-8; EMT: epithelial-mesenchymal transition; FBS:fetal bovine serum; FCM: flow cytometry; GAPDH: glyceraldehyde-3-phosphate dehydrogenase; HRP: horseradish peroxidase; lncRNA RFX3-AS1:long noncoding RNA Regulatory Factor X3- antisense RNA 1; MCM3AP-AS1: lncRNA MCM3AP antisense RNA 1; NSCLC: non-small cell lung cancer; PVDF: polyvinylidene difluoride; qRT-PCR: quantitative reverse transcription PCR; RT: room temperature; STAT3: Signal transduction and activator of transcription 3; WB: Western blot.
